# *Clostridium beijerinckii* displays a soluble [FeFe]-hydrogenase/formate dehydrogenase enzyme complex that links H_2_ and CO_2_ metabolism

**DOI:** 10.1042/BCJ20253323

**Published:** 2026-02-03

**Authors:** Sabrina Dezzani, Abdulrahman Alogaidi, Anca Pordea, Simone Morra

**Affiliations:** 1 Faculty of Engineering, University of Nottingham, Nottingham, U.K.; 2 Department of Life Sciences and Systems Biology, University of Turin, Italy; 3 University School for Advanced Studies IUSS Pavia, Italy

**Keywords:** enzymology, metalloenzymes, H_2_ metabolism, Hydrogenase, Dark fermentation

## Abstract

The production of biofuels by bacterial fermentation receives sustained attention due to the need to develop novel circular and sustainable technologies. *Clostridium beijerinckii* produces both hydrogen (H_2_) and carbon-based biofuels acetone, butanol and ethanol (ABE solvents). H_2_ metabolism in *C. beijerinckii* is complex and mostly unexplored. Seven hydrogenase genes are contained in the genome, but their exact physiological role is unknown. Here, we report on the characterisation of a novel heterotetrameric soluble enzyme complex composed of an [FeFe]-hydrogenase component stably bound to a formate dehydrogenase subunit, which we name *Cb*Fdh/Hyd. We show that the four subunits form a stable complex that can be conveniently overexpressed and purified recombinantly. *Cb*Fdh/Hyd is highly sensitive to atmospheric oxygen and displays reversible catalytic features, including H_2_ evolution, H_2_ uptake, formate oxidation and the ability to split formate into H_2_ and CO_2_ (formate hydrogen lyase activity, FHL) as well as the opposite reaction, H_2_-driven CO_2_ reduction (HDCR). *Cb*Fdh/Hyd displays functional and spectroscopic features very similar to Fdh/Hyd complexes previously described in acetogens, suggesting that this enzyme is at the basis of the previously reported unconventional ability of *C. beijerinckii* to fix CO_2_ into acetate and butyrate. *Cb*Fdh/Hyd could also represent a key player in H_2_ production metabolism by degrading formate produced from the decarboxylation of pyruvate.

## Introduction

*Clostridium beijerinckii* is a solventogenic bacterium that is known for its ability to produce acetone–butanol–ethanol (ABE) solvents by fermentation [1]. The ability to convert biomass into solvents has attracted a lot of attention on solventogenic Clostridia for their exploitation in the production of sustainable and renewable carbon-based biofuels [2,3].

Beyond its value in ABE solvent production, *C. beijerinckii* is also an excellent H_2_ producer. Biological H_2_ production is of high interest to decarbonise energy and industrial manufacturing processes [4], expanding the potential value of the species in a biorefinery context.

**Figure 1:**
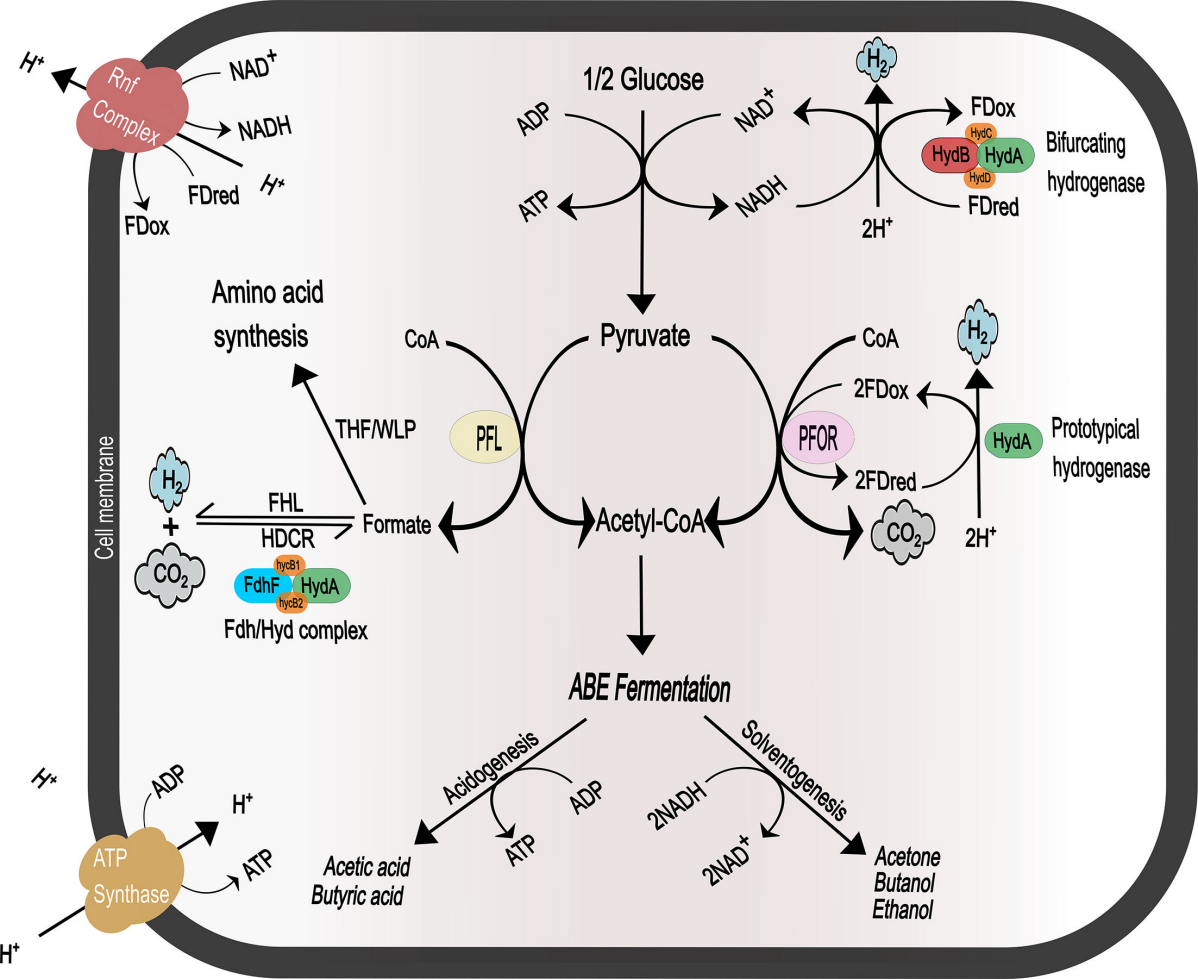
Proposed metabolic pathways in *Clostridium beijerinckii* and relevance for solvents and H_2_ metabolism. Conventionally, H_2_ is proposed to be generated as a means of re-oxidising reduced cofactors generated by glycolysis and subsequent pyruvate decarboxylation, by prototypical and bifurcating hydrogenases. In this work, we propose that a newly characterised Fdh/Hyd complex is likely involved in H_2_ metabolism of *C. beijerinckii*. FD_ox_/FD_red_ = oxidised or reduced ferredoxin. CoA, coenzyme A; PFL, pyruvate formate lyase; PFOR, pyruvate:ferredoxin oxidoreductase; FHL, formate hydrogen lyase activity; HDCR, hydrogen-dependent CO_2_ reductase activity; RNF, Rhodobacter nitrogen fixation complex; ABE, acetone–butanol–ethanol; THF/WLP, partial Wood–Ljungdahl pathway (based on tetrahydrofolate).

Several *C. beijerinckii* strains with high H_2_ production capability have been isolated from the environment and from bio-hydrogen production plants [5–9]. While H_2_ productivity is well-known for *C. beijerinckii*, the underlying molecular mechanisms that enable and control H_2_ metabolism are not fully understood. A general model for H_2_ production in dark fermentative bacteria proposes that hydrogen is produced by hydrogenases as a way of dissipating excess reducing power by re-oxidising NADH and/or ferredoxin pools generated by central energy metabolism (Figure 1) [10,11]. However, H_2_ metabolism remains poorly understood, limiting our ability to design genetically modified organisms with improved productivity [12].

The genome of *C. beijerinckii* NCIMB 8052 contains seven hydrogenase genes (including 6 [FeFe]-hydrogenases and 1 [NiFe]-hydrogenase) and a nitrogenase gene that could participate in H_2_ metabolism [13], but the exact functional role of each gene is currently unknown. All [FeFe]-hydrogenase genes (namely Cbei_0327, Cbei_1773, Cbei_1901, Cbei_3796, Cbei_4000 and Cbei_4110) have been shown to be transcribed during H_2_ production [8,9], but clarity on their role has not been provided even in detailed transcriptomics studies [1]. Among these genes, Cbei_0327 was proposed to be regulated by the redox-sensing transcriptional repressor Rex [14]. Inactivation of the bifurcating [FeFe]-hydrogenase Cbei_4110 resulted in enhanced butanol production [15], while Cbei_1773 (also known as *Cb*A5H or *Cb*HydA1) has been demonstrated to be O_2_ protected, which is an unusual feature amongst [FeFe]-hydrogenases [16–18].

Interestingly, gene Cbei_3796 could not be inactivated, suggesting that it is essential to *C. beijerinckii* survival [15]. Cbei_3796 is annotated to encode for a formate dehydrogenase (Fdh)-linked [FeFe]-hydrogenase (group A4) [19]. Group A4 [FeFe]-hydrogenases link redox processes involving H_2_, CO_2_ and formate [19], and they are crucial to acetogenesis, i.e., biological reduction of CO_2_ to acetic acid [20]. In *Acetobacterium woodii* [21] and *Thermoanaerobacter kivui* [22], group A4 [FeFe]-hydrogenases are known as hydrogen-dependent CO_2_ reductases (HDCR) because they catalyse H_2_-driven CO_2_ reduction into formic acid as part of the Wood–Ljungdahl pathway. The structure of *T. kivui* HDCR has recently shown that the enzyme forms membrane-anchored nanowires, composed of electron-conductive filaments based on a hexameric repeating unit containing one Fdh, two [FeFe]-hydrogenases and three iron sulphur-containing subunits [23].

**Figure 2:**
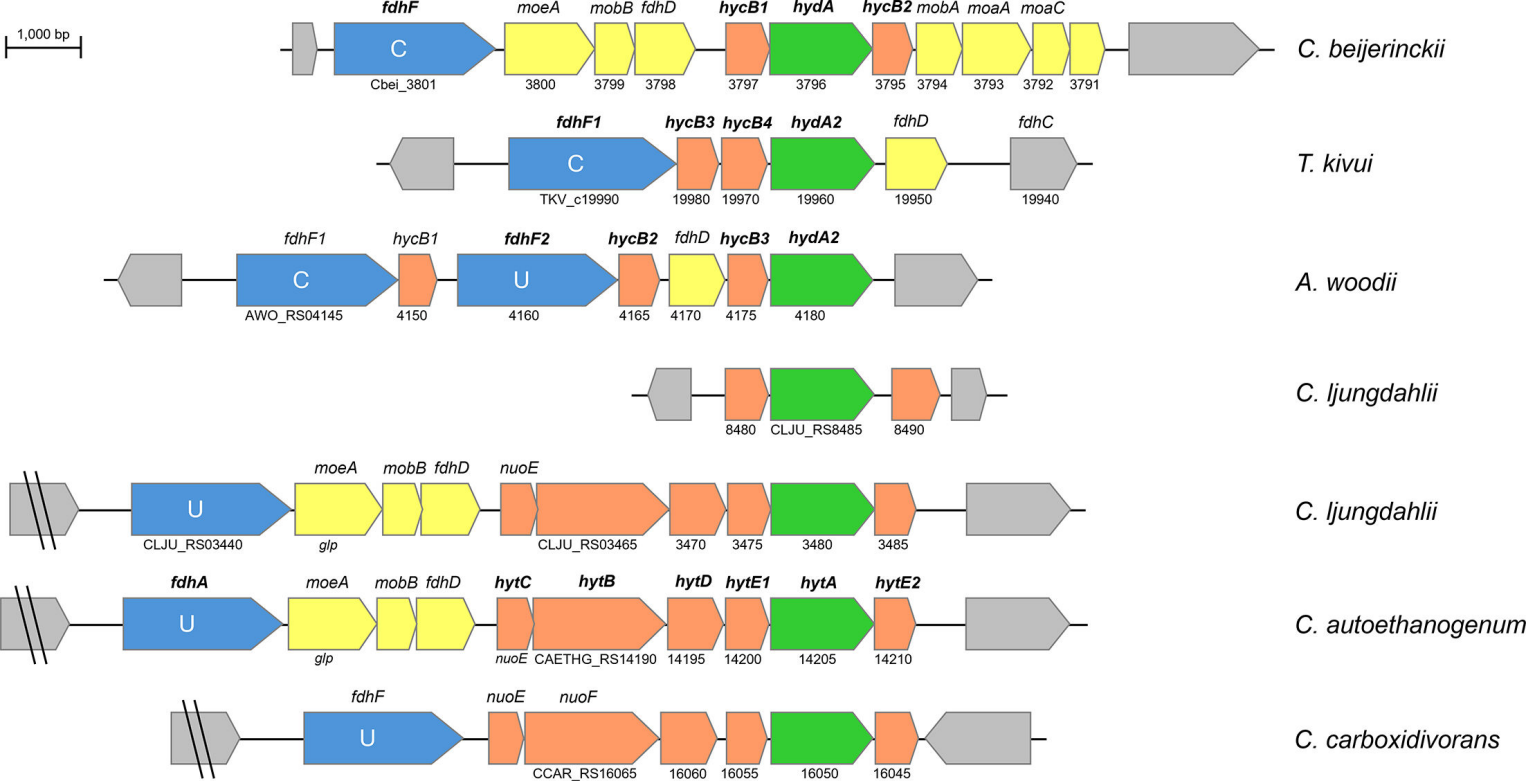
Genomic organisation of group A4 [FeFe]-hydrogenases in *C. beijerinckii* compared with selected acetogens. Colour code of predicted gene products: [FeFe]-hydrogenase catalytic subunit (green); electron transfer subunits (orange); formate dehydrogenase subunit (blue); Fdh maturation factors (yellow); flanking genes (grey). For each species, the functional name is given on top of the scheme, while the database name is given below. Database names have been abbreviated for clarity, for example, 3796 stands for Cbei_3796. Within Fdh subunits, metal co-ordination is mediated by a cysteine (**C**) or selenocysteine (**U**) residue. Gene products highlighted in bold are studied in this work in *C. beijerinckii*, while those from other species have been experimentally characterised before and shown to be part of a functional Fdh/Hyd enzyme complex [21,22,25].

While *C. beijerinckii* is not an acetogen, ^13^C-Tracer experiments have shown that it is capable of transient re-assimilation of CO_2_ and H_2_ in late log phase, i.e., at the onset of solventogenesis [24]. It has been proposed that *C. beijerinckii* assimilates CO_2_ via reversed PFOR/PFL pathway (Figure 1) where a key role is played by the formate dehydrogenase encoded by gene Cbei_3801 and by the [FeFe]-hydrogenase encoded by gene Cbei_3796 (Figure 2), which have been shown to follow similar transcriptional regulation trends [24]. To date, these genes and the corresponding enzymes have not been investigated directly, and it is not known whether they can play a functional role in *C. beijerinckii* metabolism.

In this work, we characterise *Cb*Fdh/Hyd, a new Fdh-linked [FeFe]-hydrogenase from *C. beijerinckii* NCIMB 8052. We show that the enzyme complex is composed of the heterotrimeric [FeFe]-hydrogenase Cbei_3796 linked to the Mo-containing formate dehydrogenase Cbei_3801. *Cb*Fdh/Hyd catalyses H_2_-driven reduction of carbon dioxide into formate at rates similar to HDCRs from acetogens, providing a molecular basis for CO_2_ assimilation by *C. beijerinckii*. Additionally, the new enzyme readily catalyses formate oxidation to H_2_ and CO_2_, providing the first evidence of a soluble formate hydrogen lyase (FHL) complex in *C. beijerinckii*, potentially highlighting its metabolic flexibility.

## Results

### Genomic context of group A4 heteromeric [FeFe]-hydrogenase in *C. beijerinckii*

The genomic organisation in the proximity of Cbei_3796 in *C. beijerinckii* is highly reminiscent of acetogens [26]. The *hydA* gene is flanked by genes encoding for electron transfer subunits HycB1/B2, as well as Fdh maturation factors and an upstream formate dehydrogenase gene (*fdhF*) (Figure 2). These genes are split over two separate predicted operons, encompassing Cbei_3801–3798 and Cbei_3797–3791 (Supplementary Figure S1). The presence of Fdh maturation factors in both operons suggests a potential functional linkage between the hydrogenase and the formate dehydrogenase component.

Sequence analysis of the HydA [FeFe]-hydrogenase subunit suggests it belongs to a heterotrimeric TR(M2) modular organisation [13], comprising subunits HydA/HycB1/B2. The HydA subunit displays high sequence identity to other group A4 hydrogenase subunits found in well-studied acetogens (Supplementary Table S1), such as *Thermoanaerobacter kivui* [22], *Acetobacterium woodii* [21] and *Clostridium autoethanogenum* [25]. The [4Fe4S]-containing subunits HycB1 and HycB2 also display good sequence identity to these previously characterised enzyme complexes (Supplementary Table S2). Specifically, *Cb*HycB1 sequence is more similar to *Tk*HycB4 and *Aw*HycB3, while *Cb*HycB2 sequence is more similar to *Tk*HycB3 and AwHycB2, potentially the results of genomic rearrangement. The FdhF sequence also displays high identity to formate dehydrogenases from these selected species (Supplementary Table S3). It is notable that *Cb*FdhF displays a cysteine (C) co-ordinating the predicted metal cofactor, similarly to *Tk*FdhF1, while most other formate dehydrogenases rely on selenocysteine (U).

When comparing sequence and genomic features of *C. beijerinckii* group A4 hydrogenase with existing experimental data on other hydrogenases in this group, it appears that they show common features. However, additional subunits enabling electron bifurcation HytB, HytC and HytD, as observed in *C. autoethanogenum* heptameric complex FdhA/HytA-E [25], are missing in *C. beijerinckii* gene cluster. This suggests a structural and functional similarity to non-bifurcating tetrameric complexes such as H_2_-dependent CO_2_ reductases (HDCR) from *A. woodii* [21] and *T. kivui* [22].

### A heterotrimeric [FeFe]-hydrogenase forms a stable complex with a formate dehydrogenase in *Clostridium beijerinckii*

**Figure 3:**
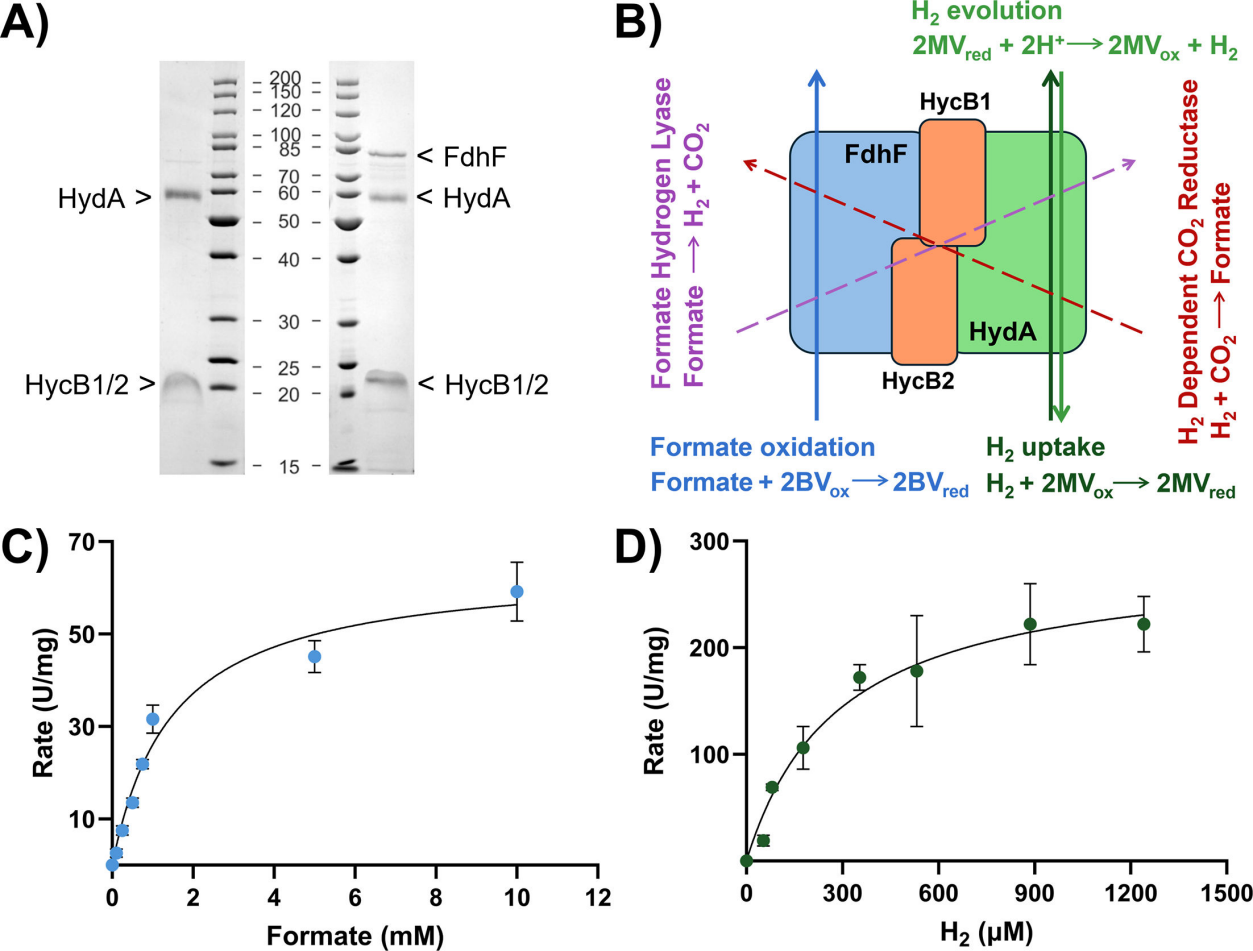
*Cb*Fdh/Hyd complex composition, reactions and substrate affinity. (**A**) SDS-PAGE analysis of the recombinant *Cb*HydA*/HycB1/B2 and *Cb*FdhF/HydA*/HycB1/B2 purified from *E. coli* BL21(DE3) ∆IscR*.* (**B**) Activities displayed by *Cb*Fdh/Hyd complex. Solid lines represent reactions occurring within individual subunit; dashed lines represent reactions requiring electron transfer across different subunits of the complex. (**C**) Michaelis–Menten plot for formate oxidation. (**D**) Michaelis–Menten plot for H_2_ uptake.

In order to provide a full enzymatic characterisation of this group A4 [FeFe]-hydrogenase, we overproduced the enzyme recombinantly in *E. coli* BL21(DE3) ∆IscR, which has proven successful for several other [FeFe]-hydrogenases before [27,28]. The heterotrimeric [FeFe]-hydrogenase encoded by genes Cbei_3797, Cbei_3796 and Cbei_3795 could be readily overexpressed and purified by affinity chromatography, yielding the *Cb*HydA/HycB1/B2 complex (Figure 3A). The heterotrimeric enzyme displayed high activity rates with a bias towards H_2_ uptake, *i.e*. oxidation of H_2_ (Table 1). All three subunits were part of the enzyme complex, as they readily co-purified when the HydA subunit was tagged (HydA*). Given the high similarity in size, it was not possible to distinguish between electron-transfer subunits HycB1 and HycB2 by SDS-PAGE. To confirm the presence of both subunits in the recombinant enzyme complex, we moved the affinity tag to subunit HycB2 (HycB2*), which is the last in the gene cluster. This complex also co-purified successfully, confirming that all three subunits were expressed and associated in a heterotrimeric complex (Supplementary Figure S2).

**Table 1:** Activity assays of samples purified from *E. coli* BL21(DE3) ∆IscR

Subunits	H_2_ evolution activity (U/mg)	H_2_ uptake activity (U/mg)	Formate oxidation activity (U/mg)
*Cb*HydA*/HycB1/B2	1,209 ± 13	8,888 ± 271	-
*Cb*HydA/HycB1/B2*	911 ± 14	6,294 ± 240	-
*Cb*FdhF/HydA*/HycB1/B2	1,316 ± 6	9,587 ± 275	Not detectable

The complexes were purified by affinity chromatography under strict anaerobic conditions. The asterisk indicated what subunit was tagged for affinity purification. Assays were conducted at 37°C in 100 mM TrisHCl supplemented with 150 mM NaCl, pH 8.

Next, we co-expressed Cbei_3801 with Cbei_3797, Cbei_3796 and Cbei_3795. All four subunits co-purified when the HydA subunit was tagged (HydA*), demonstrating that the formate dehydrogenase subunit had a strong affinity for the [FeFe]-hydrogenase component (Figure 3A). The enzyme complex *Cb*FdhF/HydA*/HycB1/B2 purified from *E. coli* BL21(DE3) ∆IscR displayed high hydrogenase activity rates, but no formate oxidation activity (Table 1).

We initially hypothesised that lack of this activity was due to the inability of *E. coli* to assemble a functional formate dehydrogenase from *C. beijerinckii*. Cloning the full gene cluster Cbei_3801-Cbei_3791, encompassing both structural genes and annotated Fdh maturation factors, did not result in productive expression of all subunits (data not shown), indicating that a different strategy was needed.

### Catalytic features of the *Cb*Fdh/Hyd complex

While *E. coli* BL21(DE3) and their derivatives are commonly used for the overproduction of iron-containing metalloenzymes, they showed severe deficiencies in the uptake of other metals, including molybdenum, which is important for the maturation of some formate dehydrogenases [29]. Given the challenges we observed in obtaining a fully functional Fdh-linked hydrogenase in *E. coli* BL21(DE3) ∆IscR, we attempted overproduction of this complex by utilising a different host strain, *E. coli* JM109(DE3) which is a K-12 derivative that can incorporate a broader variety of metals [29].

When overproduced in *E. coli* JM109(DE3), the purified enzyme complex *Cb*FdhF/HydA*/HycB1/B2, herein referred to as *Cb*Fdh/Hyd, displayed high rates of H_2_ evolution, H_2_ uptake and formate oxidation activities (Table 2), associated with the expected subunit composition (Supplementary Figure S3). The native size of the functional complex was in excess of 1.3 MDa (Supplementary Figure S4), indicating that the four subunits may be arranged in higher order structures, potentially similar to the filaments previously reported for *A. woodii* and *T. kivuii* HDCR complexes [23,30].

**Table 2:** **Activity assays of functional *Cb*Fdh/Hyd complex purified from *E. coli* JM109(DE3**)

Reaction name	Reaction	Specific activity (U/mg)
H_2_ evolution	2H^+^ + 2MV_red_^·+^ = H_2_ + 2MV_ox_^2+^	710 ± 120
H_2_ uptake	H_2_ + 2MV_ox_^2+^ = 2H^+^ + 2MV_red_^·+^	1,987 ± 147
Formate oxidation	HCOOH + 2BV_ox_^2+^ = CO_2_ + 2H^+^ + 2BV_red_^·+^	134 ± 6
Formate hydrogen lyase (FHL)	HCOOH = H_2_ + CO_2_	12 ± 1
H_2_-dependent CO_2_ reductase (HDCR)	H_2_ + CO_2_ = HCOOH	10 ± 5

The complex, including the four subunits FdhF/HydA*/HycB1/B2, was purified by affinity chromatography under strict anaerobic conditions. Assays were conducted at 37°C in 100 mM HEPES supplemented with 20 mM MgSO_4_ and 2 mM dithioerythritol, pH 7.

Similar to previously described HDCR complexes from acetogens [21,22], the formate dehydrogenase component and the hydrogenase component are expected to be within electron transfer distance, able to perform coupled reactions (Figure 3B). Indeed, the *Cb*Fdh/Hyd complex was able to oxidise formate into H_2_ and CO_2_, as well as reduce CO_2_ into formate utilising H_2_ (Table 2).

H_2_ uptake and formate oxidation activities were further studied to determine kinetic parameters by fitting methyl viologen and benzyl viologen reduction at increasing concentration of H_2_ and formate, respectively, to a Michaelis–Menten plot (Figure 3C–D). The Michaelis constants (*K*_M_) for H_2_ and formate were calculated to be 319 ± 31 µM and 1.49 ± 0.09 mM. Information on the metal content of the purified complex was obtained using inductively coupled plasma mass spectrometry (ICP-MS). The presence of molybdenum and iron was detected, thus confirming the incorporation of metals by *E. coli* JM109(DE3) and providing qualitative evidence on the nature of the Fdh metal cofactor.

### *Cb*Fdh/Hyd components are O_2_ sensitive

**Figure 4:**
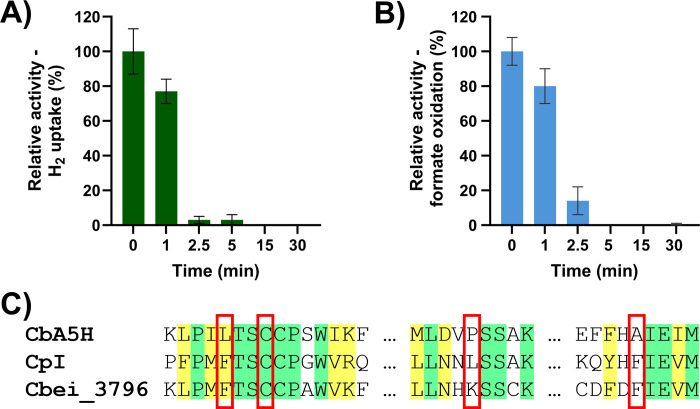
Oxygen sensitivity of the *Cb*Fdh/Hyd complex. (**A**) Residual hydrogenase activity after exposure to air. (**B**) Residual formate oxidation activity after exposure to air. (**C**) Sequence analysis of key residues proposed to enable O_2_ protection in [FeFe]-hydrogenases. *Cb*A5H is a [FeFe]-hydrogenase from *C. beijerinckii* that survives exposure to air for sustained periods of time [16,33,34]. *Cp*I is an O_2_ sensitive [FeFe]-hydrogenase from *C. pasteurianum* [35].

Most [FeFe]-hydrogenases [31] and metal-containing formate dehydrogenases [32] are irreversibly damaged by exposure to oxygen, due to their evolutionary history in anaerobic environments. However, a unique O_2_-protected [FeFe]-hydrogenase (*Cb*A5H/*Cb*HydA1) has been reported in *C. beijerinckii*, where protection is enabled by a peculiar conformational change [16–18]. We initially hypothesised that this uncommon feature could be shared with other [FeFe]-hydrogenases from the same species. However, we found that the [FeFe]-hydrogenase component of the *Cb*Fdh/Hyd displays sensitivity to air exposure, with irreversible activity loss that occurs on the timescale of minutes, as other unprotected [FeFe]-hydrogenases (Figure 4A). This behaviour is consistent with sequence analysis that reveals that three residues proposed as essential for oxygen protection in CbA5H, namely L364, P386 and A561, are not conserved in *Cb*Fdh/Hyd, which indeed shows higher similarity to conventional O_2_ sensitive [FeFe]-hydrogenases (Figure 4C).

Similarly, the formate dehydrogenase component of the complex appears to be sensitive to oxygen, with damage occurring within a few minutes of exposure (Figure 4B). This result is consistent with sequence alignment of other O₂ sensitive Fdhs (Supplementary Table S2) and the absence of any obvious features characteristic of O₂ protected formate dehydrogenases [36].

**Figure 5:**
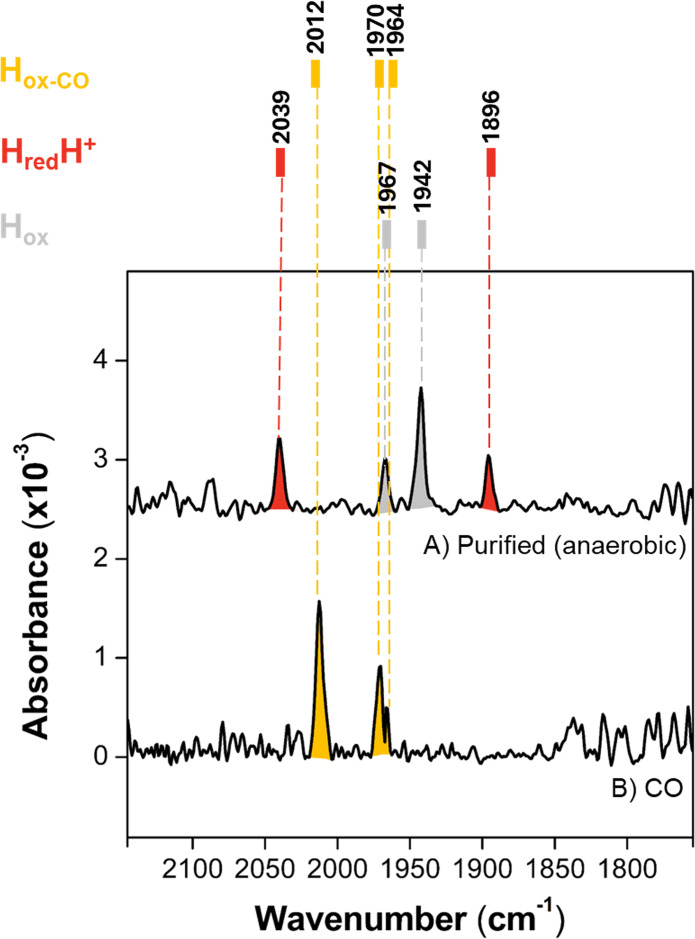
FTIR spectroscopic characterisation of *Cb*Fdh/Hyd. (**A**) The complex as purified under a N_2_/H_2_ atmosphere. (**B**) After oxidation with thionine and carbon monoxide treatment. Lower intensity peaks that are typically observed in [FeFe]-hydrogenases spectra in the regions 2050–2100 cm^-1^ and 1850–1790 cm^-1^ (Supplementary Table S4) are not distinguishable over signal noise due to low signal intensity given by limited sample concentration (approx. 0.25 mM).

*Cb*Fdh/Hyd displays Fourier Transform InfraRed (FTIR) spectroscopic features that are typical of [FeFe]-hydrogenases, revealing conventional reactivity of the catalytic metal centre H-cluster (Figure 5). The enzyme complex, as purified under a N_2_/H_2_ atmosphere, displays a mixture of oxidised (Hox) and reduced (HredH^+^) redox states (Supplementary Table S4). Upon oxidation with thionine and treatment with carbon monoxide, the Hox-CO state is formed, demonstrating that the enzyme can be CO-inhibited as other [FeFe]-hydrogenases.

## Discussion

Our results provide the first evidence that a stable and functional complex exists between a Mo-containing formate dehydrogenase and a [FeFe]-hydrogenase in *Clostridium beijerinckii*. Interestingly, the subunit composition of the *Cb*Fdh/Hyd complex is similar to previously characterised H_2_-dependent CO_2_ reductases (HDCR) from acetogens *T. kivui* and *A. woodii*, while they differ significantly from Fdh-linked enzymes observed in acetogenic species from the Clostridium genus. An Fdh-linked [FeFe]-hydrogenase has been previously characterised in *Clostridium autoethanogenum* that contains three additional subunits enabling electron bifurcation [25], which are absent in *Cb*Fdh/Hyd. Sequence analysis revealed high identity of all subunits across *C. beijerinckii* and other well characterised acetogens.

We show that beyond functionality of each individual subunit (i.e. H_2_ evolution/uptake by HydA and formate oxidation by Fdh), these are electrically connected and work co-operatively, being able to reversibly oxidise formate into H_2_ and CO_2_, or reduce CO_2_ with H_2_ yielding formate. The specific activity rates of the recombinant *Cb*Fdh/Hyd complex are in the same order of magnitude as those reported for native complexes from acetogens (Supplementary Table S5), and they are significantly higher than rates reported for a recombinant version of *A. woodii* HDCR [37]. *Cb*Fdh/Hyd activity rates are few orders of magnitude higher than those reported for *E. coli* formate hydrogen lyase complex (*Ec*FHL), although these lower rates may be due to the non-trivial isolation process of this membrane-bound complex. Interestingly, *Cb*Fdh/Hyd is not strongly biased towards a specific reaction (HDCR/FHL activity ratio ~0.8), while *Ec*FHL has a stronger bias (HDCR/FHL activity ratio ~ 3.4) [38,39].

The kinetic parameters (i.e. *K*_M_ for H_2_ and formate) are also similar to those reported for HDCRs from *A. woodii* and *T. kivui* (22, 23). Moreover, the *K*_M_ value for H_2_ is comparable with those of other [FeFe]-hydrogenases [40,41] and higher than the one reported for the [NiFe]-hydrogenase component of *Ec*FHL. This is not surprising, since *E. coli* [NiFe]-hydrogenase *K*_M_ values for hydrogen are typically lower than those of [FeFe]-hydrogenase [39]. Unfortunately, the affinity of the *Ec*FHL complex for formate has never been investigated, preventing further comparison.

The lack of O_2_ protection in *Cb*Fdh/Hyd demonstrates that *Cb*A5H’s ability to survive oxygen exposure is a built-in feature of this specific [FeFe]-hydrogenase, rather than a common feature of all *C. beijerinckii* [FeFe]-hydrogenases. This is supported by differences in sequence of residues that have been proposed to support protection from aerobic damage. Likewise, the Fdh component is O_2_ sensitive, as indicated by sequence analysis.

The preliminary spectroscopic characterisation of *Cb*Fdh/Hyd revealed that the active site H-cluster populates typical redox states that have been previously observed in other [FeFe]-hydrogenases, suggesting that the same catalytic cycle is at the basis of H_2_ catalysis [42]. Binding of CO as a competitive inhibitor is also a common feature to most [FeFe]-hydrogenases, and it has been demonstrated to be fully reversible in *A. woodii* HDCR [43].

Our kinetic data demonstrate that the *Cb*Fdh/Hyd is a cytoplasmic soluble complex that reversibly catalyses the interconversion of formate into CO_2_ and H_2_, thus potentially acting both as a formate hydrogen lyase (FHL) and a H_2_-dependent CO_2_ reductase (HDCR). The HDCR role is very likely at the basis of the reported ability of *C. beijerinckii* to capture and utilise CO_2_, when grown mixotrophically on sucrose [24]. However, this appears to be only a transient role for the enzyme. On the contrary, a role as FHL appears to be very interesting, considering that the identity of the key hydrogenase responsible for H_2_ production in *C. beijerinckii* is unknown. Previous transcriptomics work on *C. beijerinckii* 8052 did not provide any information on the transcriptional regulation of hydrogenases [44], while a study on *C. beijerinckii* NRRL B-598 concluded that it was not possible to unambiguously identify the hydrogenase gene responsible for H_2_ production [1]. However, considering that genetic inactivation of Cbei_3796 (encoding for the HydA component of the complex) is lethal [15], it is possible to propose that *Cb*Fdh/Hyd plays an important role in *C. beijerinckii* energy metabolism and H_2_ production, which is complemented by our results on the enzymatic features of the complex.

Overall, our data suggest that *Cb*Fdh/Hyd is a fully functional enzyme complex with potential physiological relevance. The main limitation of our present work is that it provides robust information on the enzyme functionality *in vitro*. Given the complexity and flexibility of *C. beijerinckii* metabolism, *Cb*Fdh/Hyd regulation and specific role *in vivo* remains to be investigated in future research. For instance, FHL functionality would require an upstream metabolic flow providing formate. It is generally accepted that the main pathway for pyruvate catabolism in *C. beijerinckii* is via PFOR, leading to oxidative decarboxylation of pyruvate into acetyl-CoA, CO_2_ and reduced ferredoxin (Figure 1) [1,3]. However, PFL genes exist in the genome, and this enzyme would convert pyruvate into acetyl-CoA and formate (Figure 1) [24,45] but evidence for such function (and its implications for *Cb*Fdh/Hyd’s role) is unclear in the existing literature.

An actively functional role for *Cb*Fdh/Hyd is also in agreement with the observation that supplementing formate to *C. beijerinckii* 8052 resulted in formate uptake and improved ABE production [45]. However, this study showed that genetic inactivation of Cbei_3801 (encoding for the Fdh component of the complex) is possible, but unfortunately H_2_ levels were not investigated [45].

The fact that *Cb*Fdh/Hyd is a soluble enzyme complex may suggest that its role in energy metabolism does not directly involve proton translocation across the plasma membrane, which has been proposed for *E. coli* FHL complex, via transmembrane HycC/D subunits [38,46]. However, given that *Cb*Fdh/Hyd forms high molecular weight complexes, it may be able to form electron-conducting filaments like *T. kivui* HDCR and these may, potentially, associate with the membrane, providing a broader scope for its function, but this remains to be investigated.

In conclusion, our work demonstrates that a group A4 [FeFe]-hydrogenase forms a stable, soluble and functional complex with a Mo-containing formate dehydrogenase in *Clostridium beijerinckii*. The complex displays high enzymatic activity for the direct and reversible conversion of formate with H_2_ and CO_2_. Our results pave the way for future more detailed mechanistic investigation of the enzyme structure and functionality. Furthermore, detailed physiologic and metabolic characterisation on *C. beijerinckii* will be required to clarify the enzyme’s role in H_2_ metabolism, and its impact on other metabolites production of industrial interest, such as ABE solvents.

## Material and methods

### Genes, plasmids and strains

All PCR amplifications were done utilising NEB Q5 High-Fidelity DNA polymerase following manufacturer’s instructions. All plasmids were constructed utilising NEB NEBuilder HiFi DNA assembly kit or T4 ligase, following manufacturer’s instructions. All plasmids were confirmed by Sanger sequencing.

Plasmid pHyd was constructed in a pET21 backbone. The partial operon containing the Cbei_3797, Cbei_3796 and Cbei_3975 genes was amplified from genomic DNA of *C. beijerinckii* NCIMB 8052 and cloned by HiFi assembly between the NdeI/XhoI restriction sites. Subsequently, the plasmid was linearised by PCR between the Cbei_3797 and Cbei_3976 genes, and an N-terminal Twin-Strep-Tag sequence followed by a TEV cleavage site was inserted in frame at the 5’ end of the Cbei_3796 gene by HiFi assembly.

Plasmid pFdh+Hyd was constructed in a pETDuet-1 backbone. Gene Cbei_3801 was amplified from genomic DNA of *C. beijerinckii* NCIMB 8052 and cloned by HiFi assembly between the NcoI/BamHI restriction sites (MCS1). A tagged version of the Cbei_3797, Cbei_3796 and Cbei_3795 genes was cut by restriction enzymes from the above-described pHyd vector and ligated between NdeI/XhoI sites in MCS2 of the Duet vector.

Plasmid pEFG, encoding for *C. acetobutylicum* maturases HydE, HydF and HydG has been previously described [33].

Plasmid maps (Supplementary Figure S5), protein sequences (Supplementary Table S6), and primer sequences (Supplementary Table S7) are provided.

*E. coli* BL21(DE3) ∆IscR was a kind gift of Prof. Thomas Happe (Ruhr-University Bochum). *E. coli* JM109(DE3) was sourced from Promega.

### Genomic and sequence analysis

Genomes utilised had the following accession IDs: *Clostridium beijerinckii* NCIMB 8052 (CP000721.1); *Thermoanaerobacter kivui* DSM 2030 (CP009170.1); *Acetobacterium woodii* DSM 1030 (NC_016894.1); *Clostridium ljungdahlii* DSM 13528 (NC_014328.1); *Clostridium autoethanogenum* DSM 10061 (NC_022592.1); *Clostridium carboxidivorans* P7 (NZ_CP011803.1). Genome visualisation and gene annotation was explored within the NCBI Graphics interface (https://www.ncbi.nlm.nih.gov/). Multiple sequence alignments and percentage identity were performed by Clustal Omega (https://www.ebi.ac.uk/jdispatcher/msa/clustalo). Operons were predicted with MicrobesOnline (http://www.microbesonline.org/operons/) [47] and Operon-mapper (https://biocomputo.ibt.unam.mx/operon_mapper/) [48]. Promoters and terminators were predicted with BPROM and FindTerm (http://www.softberry.com/).

### Enzyme overexpression and purification

Initial experiments followed standard conditions for [FeFe]-hydrogenases overproduction. pHyd or pFdh+Hyd were co-transformed with pEFG in *E. coli* BL21(DE3) ∆IscR [49]. Briefly, the strain was grown aerobically in terrific broth media supplemented with antibiotics and 2 mM ammonium ferric citrate until OD_600_ reached 0.6–0.8. Then 0.5 mM IPTG was supplemented alongside 0.5% w/v glucose, 2 mM cysteine and 25 mM sodium fumarate, and the culture was made anaerobic by sparging with argon at 20°C overnight (~22 h).

Cells were harvested anaerobically in a Don Whitley A85 anaerobic workstation operated under a nitrogen atmosphere containing small percentage of hydrogen. Cells were lysed with 1 mg/ml lysozyme and 0.5% v/v Triton X-100 in 100 mM Tris-HCl, 150 mM NaCl, pH 8, supplemented with cOmplete protease inhibitor (Roche) and Benzonase nuclease. Purification was achieved under the same anaerobic conditions by affinity chromatography, using IBA Life Sciences StrepTactin Superflow High Capacity cartridges and eluted with 5 mM desthiobiotin.

Conditions were adapted for the production of a fully functional Fdh/Hyd enzyme complex; these were the same as above but with the following differences. The host strain was *E. coli* JM109(DE3) and the growth media was terrific broth media supplemented with 5 g/l glucose, 0.5 mM ammonium ferric citrate and 1 mM sodium molybdate. Cells were induced with 0.5 mM IPTG, 2 mM cysteine, 9.4 mM sodium carbonate and 30 mM sodium formate, as previously reported for other formate dehydrogenases [50,51]. Purification was also carried out in a buffer that has been shown to be optimal for similar enzyme complexes, comprising 25 mM Tris-HCl, 20% v/v glycerol, 20 mM MgSO_4_, 300 mM NaCl, 0.5 mM dithioerythritol, pH 7.5 [21].

### Size exclusion chromatography

0.5 mL of the affinity-purified *Cb*Fdh/Hyd complex at concentration 0.75 mg/ml was tested by analytical size exclusion chromatography. A Cytiva Superdex 200 Increase 10/300 GL column was equilibrated with degassed 25 mM Tris-HCl, 20% v/v glycerol, 20 mM MgSO_4_, 300 mM NaCl, pH 7.5. The column was operated at 4°C and a flow rate of 0.35 ml/min. Although operated aerobically, care was taken to minimise air exposure. Following strict anaerobic affinity purification, the protein sample was loaded in a syringe anaerobically, and then immediately loaded in the injection loop. All tubing was washed with degassed buffer.

### Activity assays

All assays were performed anaerobically at 37°C and a minimum of technical triplicates, unless specified otherwise. Negative controls were routinely performed without enzyme addition. Reaction buffers are detailed in the relevant table captions.

The H_2_ evolution assay was performed by gas chromatography as previously described [33]. A 20 ml headspace vial containing 2 ml reaction mixture including 10 mM methyl viologen reduced with 20 mM sodium dithionite was sparged with argon. The reaction was started by adding the enzyme and the H_2_ produced was quantified on an Agilent 7820A gas chromatographer. The H_2_ uptake assay was performed spectrophotometrically as previously described [33]. A quartz cuvette containing 2.5 ml reaction mixture including 10 mM methyl viologen was sealed and sparged with hydrogen. The reaction was started by adding the enzyme and reduction of methyl viologen (ε_604_ = 13,600 M^-1^ cm^-1^) was monitored on a Shimadzu UV2600 spectrometer. The formate oxidation assay was performed spectrophotometrically adapting a method from previous reports [50]. A quartz cuvette containing 2.5 mL reaction mixture including 20 mM sodium formate and 2 mM benzyl viologen was sealed and sparged with argon. The reaction was started by adding the enzyme and reduction of benzyl viologen (ε_600_ = 7400 M^-1^ cm^-1^) was monitored on a Shimadzu UV2600 spectrometer. The FHL activity assay was performed by gas chromatography. A 20 ml headspace vial containing 2 ml reaction mixture including 50 mM sodium formate was sparged with argon. The reaction was started by adding the enzyme and the H_2_ produced was quantified on an Agilent 7820A gas chromatographer. The HDCR activity assay was performed by a coupled colorimetric enzymatic method adapted from previous reports [21]. A 20 ml headspace vial containing 1 ml reaction mixture including 50 mM sodium bicarbonate was sparged with hydrogen. After the addition of the enzyme, the reaction was incubated for 1 h, then stopped by exposing to air. The endpoint amount of formate produced was detected by adding a reaction mixture that contained 1 mM NAD^+^ and 0.75 U/ml of *Candida boidinii* formate dehydrogenase (Megazyme). Reduced NADH was quantified on a Hidex Sense microplate reader (ε_340_ = 6220 M^-1^ cm^-1^).

For the O_2_-exposure tests, the complex was exposed to air for 1–30 min; 100 µl aliquots concentrated to approx. 0.04 mg/ml were kept on ice and at intervals samples were removed and the residual activities of H_2_ uptake and formate oxidation were measured as described above. As a benchmark, both activities were assayed immediately before exposure to air.

Kinetic H_2_ uptake and formate oxidation curves were obtained as described above using varying concentrations of H_2_ and formate. Resulting rates were plotted and fitted to a Michaelis–Menten hyperbolic curve.

### FTIR spectroscopy

The *Cb*Fdh/Hyd complex was anaerobically concentrated to approx. 0.25 mM using Amicon Ultra centrifugal concentrator 30 kDa MWCO. The sample was layered in a PIKE Technologies demountable liquid transmission cell equipped with CaF_2_ windows and a 50 µm Teflon spacer. Spectra were acquired on a Bruker Tensor 27 FT-IR spectrometer at 2 cm^-1^ resolution accumulating 512 scans. The baseline was manually subtracted using OriginPro from a spline curve accounting for the water absorption in the spectral range 2150–1750 cm^-1^.

### Metal identification

Metal content in the enzyme was determined by inductively coupled plasma mass spectrometry (ICP-MS) with a Thermo Fisher iCAP-Q instrument, after digestion of the protein in nitric acid (trace metal grade, Fisher Scientific) as previously described [52].

## Supplementary material

online supplementary material 1.

## Data Availability

All data that support the findings in this study are available within the paper (main and supplementary sections).

## Open Access

Open access for this article was enabled by the participation of the University of Nottingham in an all-inclusive Read & Publish agreement with Portland Press and the Biochemical Society under an agreement with JISC.

## Abbreviations

ABE: acetone–butanol–ethanol
FHL: formate hydrogen lyase
Fdh: formate dehydrogenase
HDCR: H_2_-dependent CO_2_ reductase
Hyd: hydrogenase
PFL: pyruvate formate lyase
PFOR: pyruvate:ferredoxin oxidoreductase
