# Correction: *Clostridium beijerinckii* displays a soluble [FeFe]-hydrogenase/formate dehydrogenase enzyme complex that links H_2_ and CO_2_ metabolism

**DOI:** 10.1042/BCJ20253323_COR

**Published:** 2026-08-12

**Authors:** Sabrina Dezzani, Abdulrahman Alogaidi, Anca Pordea, Simone Morra

**Affiliations:** 1 Faculty of Engineering, University of Nottingham, Nottingham, U.K.; 2 Department of Life Sciences and Systems Biology, University of Turin, Italy; 3 University School for Advanced Studies IUSS Pavia, Italy

**Keywords:** Dark fermentation, enzymology, H2 metabolism, Hydrogenase, metalloenzymes

The authors of the original article ‘*Clostridium beijerinckii* displays a soluble [FeFe]-hydrogenase/formate dehydrogenase enzyme complex that links H_2_ and CO_2_ metabolism’, DOI: 10.1042/BCJ20253323, would like to correct Figure 3.

The authors state that an error in the conversion factors was applied when calculating the x-axis of panel D, meaning that values originally presented were larger than they should be. This subsequently impacted the calculated Michaelis constant for H_2_.

**Figure 3:**
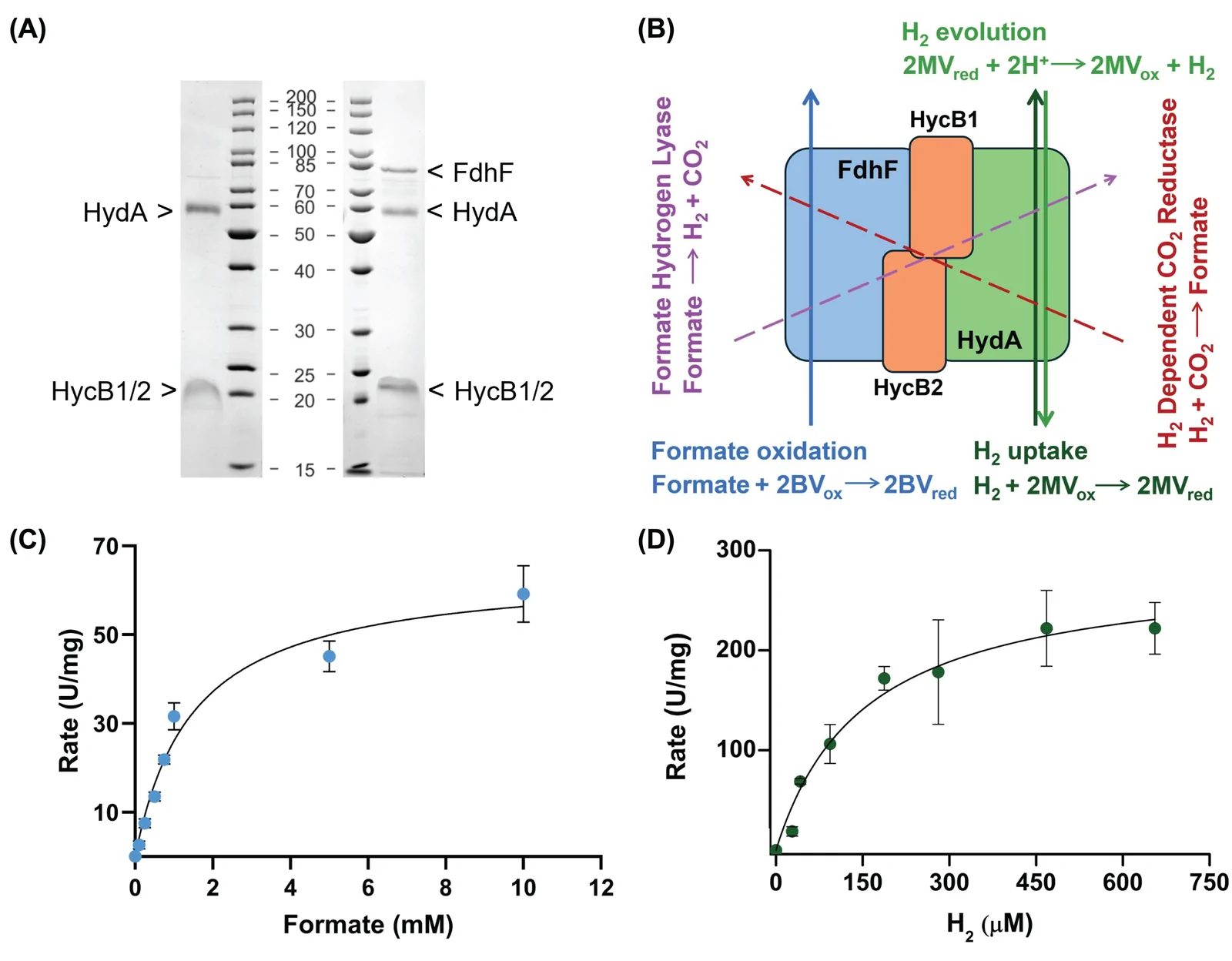
CbFdh/Hyd complex composition, reactions and substrate affinity **(A)** SDS-PAGE analysis of the recombinant CbHydA*/HycB1/B2 and CbFdhF/HydA*/HycB1/B2 purified from E. coli BL21(DE3) ∆IscR. **(B)** Activities displayed by CbFdh/Hyd complex. Solid lines represent reactions occurring within individual subunit; dashed lines represent reactions requiring electron transfer across different subunits of the complex. **(C)** Michaelis–Menten plot for formate oxidation. **(D)** Michaelis–Menten plot for H2 uptake.

The requested correction has been assessed and approved by the Editorial Board. The authors declare that these corrections do not change the results or conclusions of their paper.

Corrected text in the fourth paragraph of the section ‘Catalytic features of the CbFdh/Hyd complex’ therefore reads as follows:

‘The Michaelis constants (KM) for H_2_ and formate were calculated to be 154 ± 36 μM and 1.49 ± 0.09 mM’.

The corrected version of Figure 3 is presented here.

