# Supplementary material for: Clostridium beijerinckii displays a soluble [FeFe]-hydrogenase/formate dehydrogenase enzyme complex that links H2 and CO2 metabolism
Source: Biochem J. 2026 Feb 3;483(2):177–89. doi: 10.1042/BCJ20253323 (PMC12905484; doi:10.1042/BCJ20253323)
Supplement: online supplementary material 1. [file bcj-483-2-BCJ20253323-s001.docx]

***Clostridium beijerinckii* displays a soluble [FeFe]-hydrogenase/formate dehydrogenase enzyme complex that links H_2_ and CO_2_ metabolism**

Sabrina Dezzani, Abdulrahman Alogaidi, Anca Pordea, Simone Morra

**Supplementary Data**

**Table S1.** Hydrogenase subunit protein sequence identity matrix.

|  | **Awo** | **CljuTR** | **Cbei** | **Tkv** | **Ccar** | **CljuHEP** | **Caethg** |
| --- | --- | --- | --- | --- | --- | --- | --- |
| **Awo** | 100 | 61.56 | 60.39 | 65.05 | 65.20 | 64.04 | 64.04 |
| **CljuTR** | 61.56 | 100 | 62.56 | 66.67 | 64.69 | 66.01 | 65.79 |
| **Cbei** | 60.39 | 62.56 | 100 | 65.58 | 64.41 | 66.09 | 65.87 |
| **Tkv** | 65.05 | 66.67 | 65.58 | 100 | 69.78 | 67.90 | 67.68 |
| **Ccar** | 65.20 | 64.69 | 64.41 | 69.78 | 100 | 75.43 | 75.65 |
| **CljuHEP** | 64.04 | 66.01 | 66.09 | 67.90 | 75.43 | 100 | 99.78 |
| **Caethg** | 64.04 | 65.79 | 65.87 | 67.68 | 75.65 | 99.78 | 100 |

List of acronyms and sequence accession ID used:

Awo = *A. woodii* HydA2 (WP_014355220.1). CljuTR = *C. ljungdahlii* CLJU_RS8485 (WP_013238388.1). Cbei = *C. beijerinckii* HydA (ABR35913.1). Tkv = *T. kivui* HydA2 (AIS53140.1). Ccar = *C. carboxidivorans* CCAR_RS16050 (WP_048030161.1). CljuHEP = *C. ljungdahlii* CLJU_RS03480 (WP_013237377.1). Caethg = *C. autoethanogenum* HytA (WP_023162857.1).

**Table S2.** Electron transfer subunits protein sequence identity matrix.

|  | **CljuTR2** | **AwoB1** | **AwoB2** | **CbeiB2** | **CljuE2** | **CaethgE2** | **CcarE2** | **TkvB3** | **AwoB3** | **CbeiB1** | **TkvB4** | **CljuTR1** | **CljuE1** | **CaethgE1** | **CcarE1** |
| --- | --- | --- | --- | --- | --- | --- | --- | --- | --- | --- | --- | --- | --- | --- | --- |
| **CljuTR2** | 100 | 16.67 | 17.89 | 17.27 | 16.98 | 16.98 | 17.70 | 11.11 | 15.69 | 17.31 | 17.76 | 17.86 | 14.00 | 14.00 | 18.00 |
| **AwoB1** | 16.67 | 100 | 82.39 | 49.43 | 45.93 | 45.93 | 46.59 | 41.57 | 35.93 | 35.54 | 37.43 | 36.78 | 36.63 | 36.63 | 34.88 |
| **AwoB2** | 17.89 | 82.39 | 100 | 50.00 | 44.19 | 44.19 | 45.71 | 43.64 | 35.93 | 36.75 | 36.84 | 39.88 | 39.53 | 39.53 | 37.79 |
| **CbeiB2** | 17.27 | 49.43 | 50.00 | 100.00 | 51.35 | 51.35 | 52.13 | 43.58 | 37.43 | 39.23 | 39.78 | 43.09 | 44.38 | 44.38 | 42.70 |
| **CljuE2** | 16.98 | 45.93 | 44.19 | 51.35 | 100.00 | 100.00 | 56.68 | 41.24 | 37.29 | 40.88 | 43.78 | 42.16 | 40.00 | 40.00 | 43.43 |
| **CaethgE2** | 16.98 | 45.93 | 44.19 | 51.35 | 100.00 | 100.00 | 56.68 | 41.24 | 37.29 | 40.88 | 43.78 | 42.16 | 40.00 | 40.00 | 43.43 |
| **CcarE2** | 17.70 | 46.59 | 45.71 | 52.13 | 56.68 | 56.68 | 100.00 | 44.81 | 38.89 | 43.65 | 41.40 | 43.68 | 42.70 | 42.70 | 43.82 |
| **TkvB3** | 11.11 | 41.57 | 43.64 | 43.58 | 41.24 | 41.24 | 44.81 | 100.00 | 39.18 | 39.31 | 47.46 | 43.09 | 41.18 | 41.18 | 44.12 |
| **AwoB3** | 15.69 | 35.93 | 35.93 | 37.43 | 37.29 | 37.29 | 38.89 | 39.18 | 100.00 | 45.71 | 51.91 | 45.86 | 44.94 | 44.94 | 46.63 |
| **CbeiB1** | 17.31 | 35.54 | 36.75 | 39.23 | 40.88 | 40.88 | 43.65 | 39.31 | 45.71 | 100.00 | 52.17 | 52.46 | 44.83 | 44.83 | 48.28 |
| **TkvB4** | 17.76 | 37.43 | 36.84 | 39.78 | 43.78 | 43.78 | 41.40 | 47.46 | 51.91 | 52.17 | 100.00 | 56.38 | 48.62 | 48.62 | 53.04 |
| **CljuTR1** | 17.86 | 36.78 | 39.88 | 43.09 | 42.16 | 42.16 | 43.68 | 43.09 | 45.86 | 52.46 | 56.38 | 100.00 | 46.41 | 46.41 | 58.01 |
| **CljuE1** | 14.00 | 36.63 | 39.53 | 44.38 | 40.00 | 40.00 | 42.70 | 41.18 | 44.94 | 44.83 | 48.62 | 46.41 | 100.00 | 100.00 | 64.13 |
| **CaethgE1** | 14.00 | 36.63 | 39.53 | 44.38 | 40.00 | 40.00 | 42.70 | 41.18 | 44.94 | 44.83 | 48.62 | 46.41 | 100.00 | 100.00 | 64.13 |
| **CcarE1** | 18.00 | 34.88 | 37.79 | 42.70 | 43.43 | 43.43 | 43.82 | 44.12 | 46.63 | 48.28 | 53.04 | 58.01 | 64.13 | 64.13 | 100.00 |

List of acronyms and sequence accession ID used:

CljuTR2 = *C. ljungdahlii* CLJU_RS8490 (WP_013238389.1). AwoB1 = *A. woodii* HycB1 (WP_014355215.1). AwoB2 = *A. woodii* HycB2 (WP_014355217.1). CbeiB2 = *C. beijerinckii* HycB2 (ABR35912.1). CljuE2 = *C. ljungdahlii* CLJU_RS03485 (WP_013237378.1). CaethgE2 = *C. autoethanogenum* HytE2 (WP_013237378.1). CcarE2 = *C. carboxidivorans* CCAR_RS16045 (WP_007063271.1). TkvB3 = *T. kivui* HycB3 (AIS53142.1). AwoB3 = *A. woodii* HycB3 (WP_014355219.1). CbeiB1 = *C. beijerinckii* HycB1 (ABR35914.1). TkvB4 = *T. kivui* HycB4 (AIS53141.1). CljuTR1 = *C. ljungdahlii* CLJU_RS8480 (WP_013238387.1). CljuE1 = *C. ljungdahlii* CLJU_RS03475 (WP_013237376.1). CaethgE1 = *C. autoethanogenum* HytE1 (WP_013237376.1). CcarE1 = *C. carboxidivorans* CCAR_RS16055 (WP_007063269.1).

**Table S3.** Formate dehydrogenase subunit protein sequence identity matrix.

|  | **Tkv** | **AwoF1** | **AwoF2** | **Cbei** | **Ccar** | **CljuHEP** | **Caethg** |
| --- | --- | --- | --- | --- | --- | --- | --- |
| **Tkv** | 100 | 46.40 | 45.20 | 47.80 | 49.65 | 47.93 | 47.79 |
| **AwoF1** | 46.40 | 100 | 79.78 | 67.46 | 66.43 | 65.44 | 65.30 |
| **AwoF2** | 45.20 | 79.78 | 100 | 69.42 | 70.07 | 68.83 | 68.69 |
| **Cbei** | 47.80 | 67.46 | 69.42 | 100 | 75.60 | 72.64 | 72.50 |
| **Ccar** | 49.65 | 66.43 | 70.07 | 75.60 | 100 | 84.63 | 84.49 |
| **CljuHEP** | 47.93 | 65.44 | 68.83 | 72.64 | 84.63 | 100 | 99.86 |
| **Caethg** | 47.79 | 65.30 | 68.69 | 72.50 | 84.49 | 99.86 | 100 |

List of acronyms and sequence accession ID used:

Tkv = *T. kivui* FdhF1 (AIS53143.1). AwoF1 = *A. woodii* FdhF1 (WP_014355214.1). AwoF2 = *A. woodii* FdhF2 (WP_014355216.1). Cbei = *C. beijerinckii* FdhF (ABR35918.1). Ccar = *C. carboxidivorans* FdhF (WP_081457856.1). CljuHEP = *C. ljungdahlii* CLJU_RS03440 (WP_013237369.1). Caethg = *C. autoethanogenum* FdhA (WP_081650146.1).

**Table S4.** Comparison of FTIR peak positions.

| **Redox state** | **Enzyme** | **FTIR peaks** | **Reference** |
| --- | --- | --- | --- |
| Hox | *Cb*Fdh/Hyd | ND, ND, 1967, 1942, ND | This work |
|  | *Cb*A5H | 2091, 2080, 1964, 1940, 1800 | [1] |
|  | *Cp*I | 2082, 2071, 1970, 1947, 1800 | [2, 3] |
|  | *Cr*HydA1 | 2088, 2072, 1963, 1939, 1803 | [4] |
|  | *Dd*H | 2094, 2079, 1965, 1940, 1802 | [5, 6] |
|  | *Tm*HydABC | 2090, 2076, 1964, 1939, 1802 | [7] |
|  | *Me*HydA | 2087, 2079, 1964, 1937, 1803 | [8] |
|  | *Tam*HydS | 2083, 2073, 1971, 1947, 1788 | [9] |
| Hox-CO | *Cb*Fdh/Hyd | ND, ND, 2012, 1970, 1964, ND | This work |
|  | *Cb*A5H | 2094, 2090, 2016, 1971, 1963, 1807 | [1] |
|  | *Cp*I | 2090, 2076, 2015, 1973, 1969, 1807 | [2, 10] |
|  | *Cr*HydA1 | 2092, 2084, 2013, 1970, 1964, 1810 | [11] |
|  | *Dd*H | 2096, 2089, 2017, 1972, 1963, 1812 | [5, 6] |
|  | *Tm*HydABC | 2092, 2085, 2008, 1970, 1961, 1807 | [7] |
|  | *Me*HydA | 2090, 2090, 2013, 1969, 1955, 1804 | [8] |
|  | *Tam*HydS | 2088, 2082, 2026, 1978, 1966, 1786 | [9] |
| HredH+ | *Cb*Fdh/Hyd | ND, 2040, ND, 1896, ND | This work |
|  | *Cb*A5H | 2075, 2041, 1916, 1893, (1810) | [12] |
|  | *Cp*I | 2054, 2039, 1915, 1895, ND | [10] |
|  | *Cr*HydA1 | 2071, 2032, 1968, 1914, 1891 | [4] |
|  | *Dd*H | 2079, 2040, 1964, 1915, 1894 | [5, 6] |
|  | *Tm*HydABC | 2075, 2037, 1956, 1919, 1887 | [7] |
|  | *Me*HydA | 2069, 2041, 1956, 1916, 1891 | [8] |
|  | *Tam*HydS | 2064, 2032, 1972, 1922, 1896 | [9] |
| Hinact | *Cb*Fdh/Hyd | Not observed | This work |
|  | *Cb*A5H | 2107, 2080, 2011, 1992, 1840 | [1] |
|  | *Cp*I | Not observed | [5] |
|  | *Cr*HydA1 (Na_2_S) | Observed but peaks not detailed. | [5] |
|  | *Dd*H | 2106, 2086, 2007, 1983, 1847 | [5, 6] |

**Table S5.** Comparison of specific activity rates and K_M_ values for *Cb*Fdh/Hyd and HDCR/FHL complexes available in previous literature. “Native” denotes enzyme preparations purified from the original organism. “Recombinant” denotes enzyme preparations obtained by heterologous overexpression in *E. coli*. T refers to the temperature of the assays. * highlights formate oxidation assays done with methyl viologen as electron acceptor rather than benzyl viologen. nd = not determined. *Tk*HDCR = *T. kivui* HDCR complex. *Aw*HDCR = *A. woodii* HDCR complex. *Ec*FHL = *E. coli* FHL complex. *Ca*FdhA/HytA-E = *C. autoethanogenum* complex.

| **Complex** | **Source** | **T**  **(°C)** | **H_2_ uptake**  **(U/mg)** | **Formate oxidation**  **(U/mg)** | **K_M_ H_2_**  **(µM)** | **K_M_ Formate**  **(mM)** | **Formate → H_2_ + CO_2_**  **(U/mg)** | **H_2_ + CO_2_ → Formate**  **(U/mg)** | **Reference** |
| --- | --- | --- | --- | --- | --- | --- | --- | --- | --- |
| *Cb*Fdh/Hyd | Recombinant | 37 | 1,987 ± 147 | 134 ± 6 | 319 ± 31 | 1.49 ± 0.09 | 12 ± 1 | 10 ± 5 | This work |
| *Tk*HDCR | Native | 60 | 14,400 | 455 * | 130 | 0.55 | 450 | 900 | [13] |
| *Aw*HDCR | Native | 30 | 10,800 | 600 * | 125 ± 31 | 1 ± 0.3 | 14 | 10 | [14] |
| *Aw*HDCR | Recombinant | 30 | nd | nd | nd | nd | 1.4 | 0.53 | [15] |
| *Ec*FHL | Native | 37 | 2.44 ± 0.62 | 0.82 ± 0.04 | 34 | nd | 0.03 | 0.103 | [16, 17] |
| *Ca*FdhA/HytA-E | Native | 37 | 18,000 | 170 * | nd | nd | 40 | 41 | [18] |

**Table S6.** Protein sequences expressed by the plasmids constructed in this study

The Twin-Step-Tag sequence is underlined. The TEV cleavage site is double underlined.

| >Cbei_3801 (FdhF, 80.2 kDa)  MEKKVLTVCPYCGSGCNLYLVVKDGKIVRAEPANGDNNEGKLCLKGYYGWDFLNDPQILTSRIKKPMIRKNGELEEVSWDEAIKFTAENLMKIKAKYGPDAIMGTGSARGPGNEPNYVMQKFMRAVIGTNNIDHCARVCHGPSVAGLTYSLGDGAMSNSIPEIEDSDVLFIFGYNPAETHPIVARRIVKAKEKGAKVIVTDPRKTDVVRLSDLWLQLKGGTNMALVNAFGNVLINEDLYDHDYVEKYTEDFEKYKAEVEKYTPEYAEKITGVKADYIRKAMRTYAKAKNATILYGMGVCQFSQAVDVVRGLASIALLTGNFARPSVGIGPVRGQNNVQGTCDMGTIPNNYPGYQLVTDKKVQEKFEKAWGVELSDKNGYFLTQVPELVLKEDKIKAYYIFGEDPVQSDPNAAELREALDKIEFIVVQDIFMNKTALHADVILPSTSWGEHDGVYSSADRGFQRIRKAVEPLGDVKTDWEIISAVATAMGYPMNYKNTEEIWDEMRSLCPKFAGASYKKIEEQGIVRWPCPTEDHKGTSYLYEGNKFTTPSGKGRLFACEWRPPVELTDEEYPLSLSTVREVGHYSVRTMTGNCRALQKLADEPGFIQMSIEDAEELNIKDQELVRITSRRGSVLSRALITERVKKGSTFMTYQWWIGACNELTNDSLDPISKTPEFKYCAIKVERIEDQKSAEEYIITEYENIRKKMRITSEK |
| --- |
| >Cbei_3797 (HycB1, 20.3 kDa)  MKSCNSFIVGDANKCVGCKACEIACFKAHNESVTVGNIQTPIISRIHVIKEKDFTVPVQCRHCENAPCAKVCPINAIKNEDNAIIIDEEICIGCKACAVACPFGAIEMGTKYKDGKAVMQNVQKELFEEVLEEKETKVAYKCDLCKEQGEPACVKACPKDALKLFDVIEEKRIRNIRAVSNLNL |
| >Cbei_3796 (tagged HydA, 56.3 kDa)  MASAWSHPQFEKGGGSGGGSGGSAWSHPQFEKGGGSGGGSGGGSENLYFQGDIGFVNIDKELCTGCQQCVEVCPVNAIQGKKGQPQNIDYDVCVSCGQCIQVCNSYGFENRENSHLIEEKRRDRGVLESVKEPVFAAFNKGNAAKVKEALHDEELFTIVQCAPAVRVSLGEEFGLKAGSLTAGKMAAALRRLGFNRVYDTNFGADLTIMEEGSELIKRVTEGGELPMFTSCCPAWVKFMEQSYPELLNHLSSCKSPQQMAGTIFKTYGAKIDKVNPKKIYNVAIMPCTCKQFECDREEMQDSGFKDVDIVITTREFAQLIRDNEIDFKNLKDEEFDLPLGSYTGAGNIFGVTGGVMEAALRSGYEMLTKKSIPNLELNFVRGSEGIRVAEVKLPKITLKVAVVSGLKNVVQILEDIKEGKCDFDFIEVMTCPEGCVSGGGQPKFILDIDRRNALVSRKKGIYKHDSELEIRKSHENPFIKKLYEEFLIEPLGEKSHHLLHTKFVSRKKEEI |
| >Cbei_3796 (untagged HydA, 51.5 kDa)  MDIGFVNIDKELCTGCQQCVEVCPVNAIQGKKGQPQNIDYDVCVSCGQCIQVCNSYGFENRENSHLIEEKRRDRGVLESVKEPVFAAFNKGNAAKVKEALHDEELFTIVQCAPAVRVSLGEEFGLKAGSLTAGKMAAALRRLGFNRVYDTNFGADLTIMEEGSELIKRVTEGGELPMFTSCCPAWVKFMEQSYPELLNHLSSCKSPQQMAGTIFKTYGAKIDKVNPKKIYNVAIMPCTCKQFECDREEMQDSGFKDVDIVITTREFAQLIRDNEIDFKNLKDEEFDLPLGSYTGAGNIFGVTGGVMEAALRSGYEMLTKKSIPNLELNFVRGSEGIRVAEVKLPKITLKVAVVSGLKNVVQILEDIKEGKCDFDFIEVMTCPEGCVSGGGQPKFILDIDRRNALVSRKKGIYKHDSELEIRKSHENPFIKKLYEEFLIEPLGEKSHHLLHTKFVSRKKEEI |
| >Cbei_3795 (tagged HycB2, 25.2 kDa)  MASAWSHPQFEKGGGSGGGSGGSAWSHPQFEKGGGSGGGSGGGSENLYFQGNNFVIANPKRCIGCRTCEAACVVAHSEENILIQSKDKVNFNPRLKVIKTADVSAPIQCRHCENAPCANACPNGSIINKDGVVLINKDTCIGCKSCAIVCPFGAIDIIVEHKAGEKVIQKGLMCDKDGKLEHKERLVANKCDLCIGRENGPACVEVCPTEALRLVESKIIDKDISEKRKNAAANLVNIL |
| >Cbei_3795 (untagged HycB2, 20.5 kDa)  MNNFVIANPKRCIGCRTCEAACVVAHSEENILIQSKDKVNFNPRLKVIKTADVSAPIQCRHCENAPCANACPNGSIINKDGVVLINKDTCIGCKSCAIVCPFGAIDIIVEHKAGEKVIQKGLMCDKDGKLEHKERLVANKCDLCIGRENGPACVEVCPTEALRLVESKIIDKDISEKRKNAAANLVNIL |

**Table S7.** Primers list

| **Primer ID** | **Primer sequence (5’-3’)** | **Scope** |
| --- | --- | --- |
| gDNA_Hyd_F | CTTGGGAGTGCCTTGAATGT | Amplify gene cluster Cbei_3797, Cbei_3976, Cbei_3795 from gDNA |
| gDNA_Hyd_R | ATCAAAGCCCATTCGTGAAC |  |
| gDNA_Fdh_F | CGTTGAAATAAACAGCCCTTG | Amplify gene Cbei_3801 from gDNA |
| gDNA_Fdh_R | TTGCTTGTGCCTTATCATTTTC |  |
| Hyd_Clone_F | CTTTAAGAAGGAGATATACATATGAAAAGTTGTAATTCTTTCATTG | Clone Cbei_3797, Cbei_3796, Cbei_3795 in pET21 |
| Hyd_Clone_R | AGTGGTGGTGGTGGTGGTGCTCGAGTTATAATATATTTACTAAATTAGC |  |
| Fdh_Clone_F | ACTTTAAGAAGGAGATATACCATGGAAAAGAAAGTGTTAACG | Clone Cbei_3801 in MCS1 of pETDuet-1 |
| Fdh_Clone_R | GCGCGCCGAGCTCGAATTCGGATCCCTATTTTTCAGATGTTATTCTC |  |
| 3796_Vec_F | ttttcaaggtGATATTGGTTTTGTAAATATTGATAAAG | Insert Tag at N-terminus of Cbei_3796 |
| 3796_Vec_R | cgcttgccatAAATTAAATTCCTCTCTCTTATAAGTTTAAG |  |
| 3796_Tag_F | aatttaatttATGGCAAGCGCATGGTCAC |  |
| 3796_Tag_R | aaccaatatcACCTTGAAAATACAGATTTTCGCTGC |  |
| 3795_Vec_F | ttttcaaggtAATAATTTTGTTATAGCAAATCCAAAAAG | Insert Tag at N-terminus of Cbei_3795 |
| 3795_Vec_R | cgcttgccatTTATATTTCCTCCTTCTTTCTC |  |
| 3795_Tag_F | ggaaatataaATGGCAAGCGCATGGTCAC |  |
| 3795_Tag_R | caaaattattACCTTGAAAATACAGATTTTCGCTGC |  |


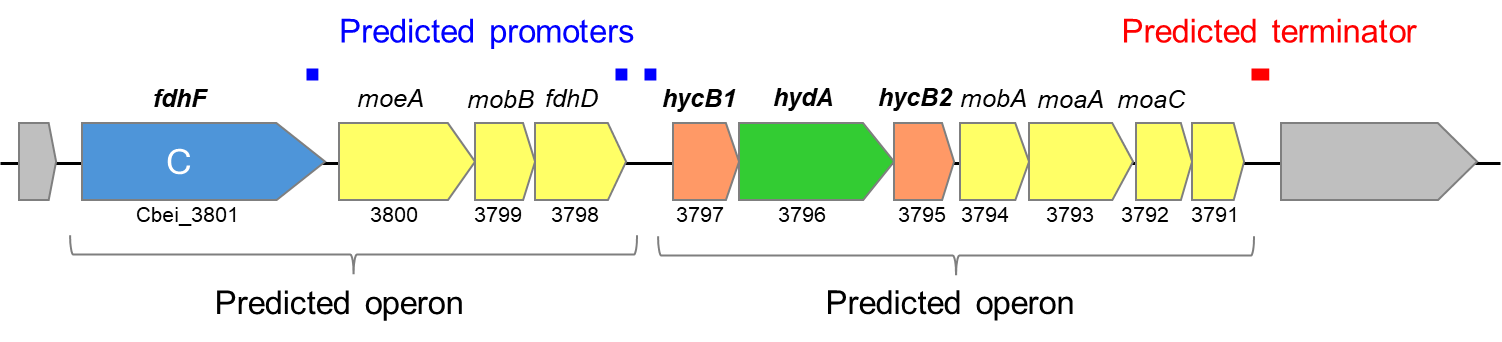


**Figure S1.** Analysis of predicted operon structure.


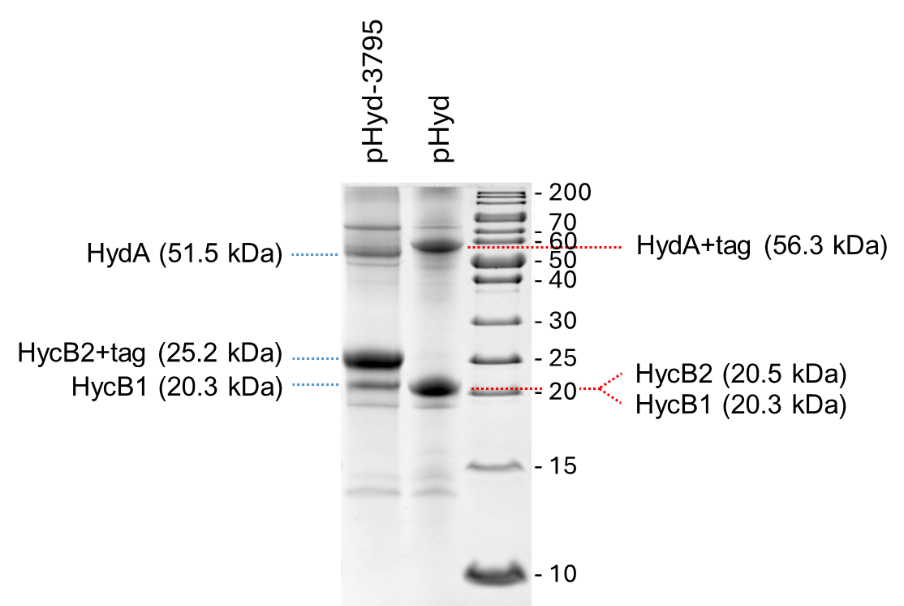


**Figure S2.** Tricine-SDS-PAGE analysis of subunits in the heterotrimeric [FeFe]-hydrogenase complex. Samples were run on 15% acrylamide gel prepared with the Tricine chemistry, optimised for the detection of small proteins [19].


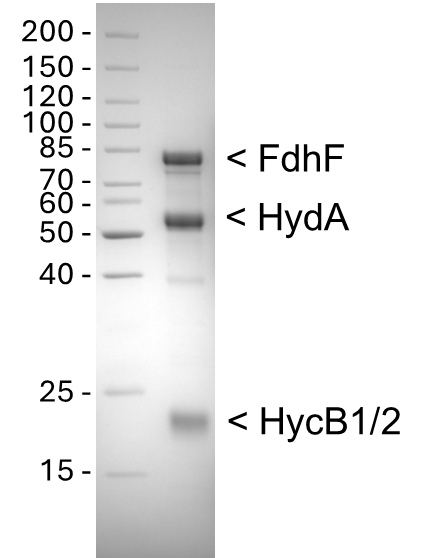


**Figure S3.** SDS-PAGE analysis of the active *Cb*Fdh/Hyd complex purified from *E. coli* JM109(DE3).


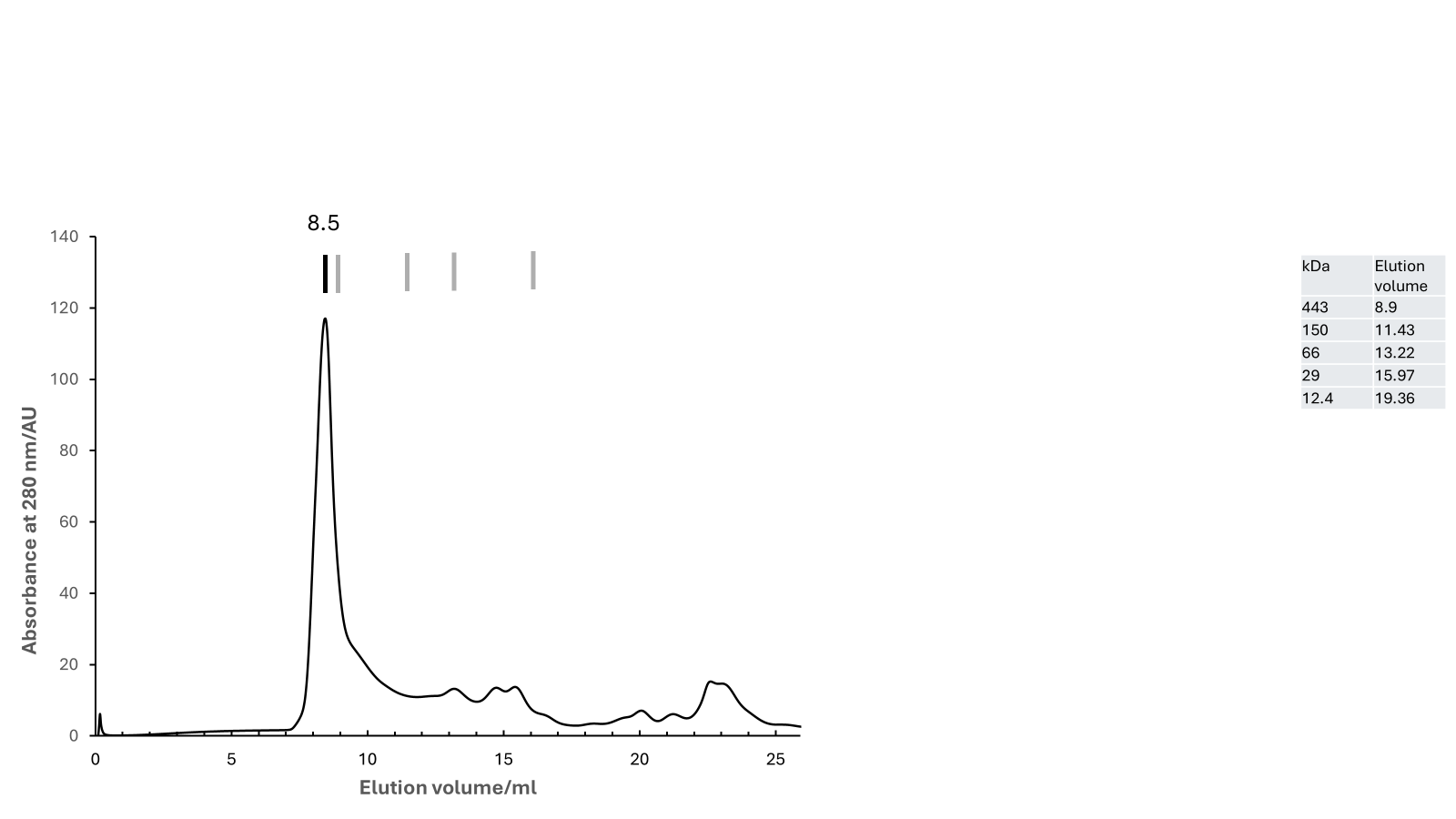


**Figure S4.** Size exclusion chromatography of 375 µg *Cb*Fdh/Hyd complex purified from *E. coli* JM109(DE3). Conditions: Cytiva Superdex 200 Increase 10/300 GL equilibrated with 25 mM Tris-HCl, 20% v/v glycerol, 20 mM MgSO_4_, 300 mM NaCl, pH 7.5. The black bar indicates the elution volume of the target protein (8.5 mL), corresponding to the column void volume (exclusion limit 1.3 MDa). The grey bars indicate the elution volume of protein standards which were: apoferritin (449 kDa) at 8.9 mL, alcohol dehydrogenase (150 kDa) at 11.4 mL, bovine serum albumin (66 kDa) at 13.2 mL, and carbonic anhydrase (29 kDa) at 16 mL.


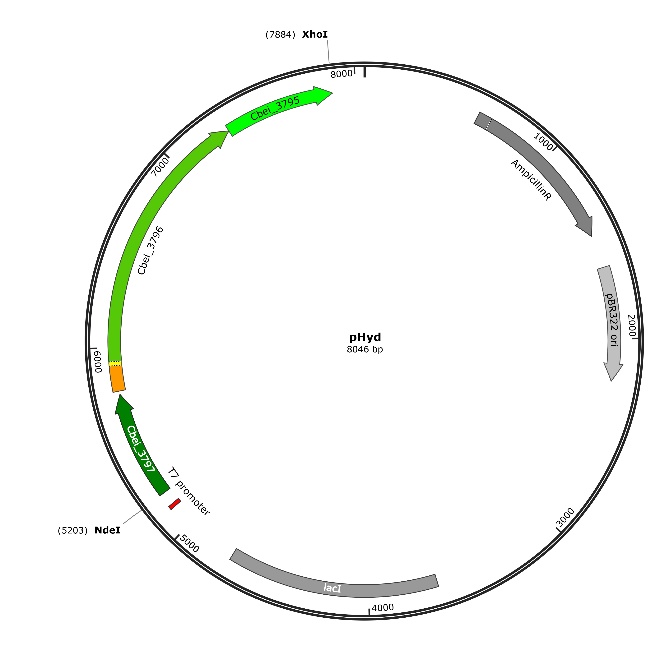

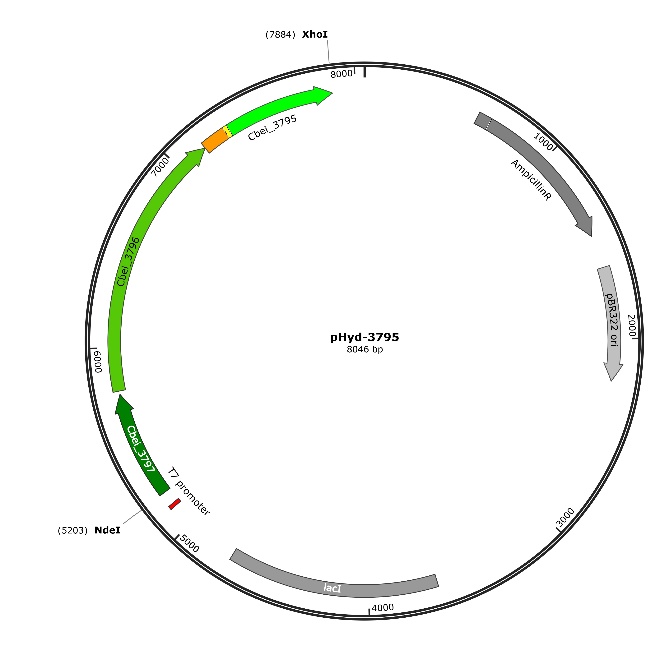


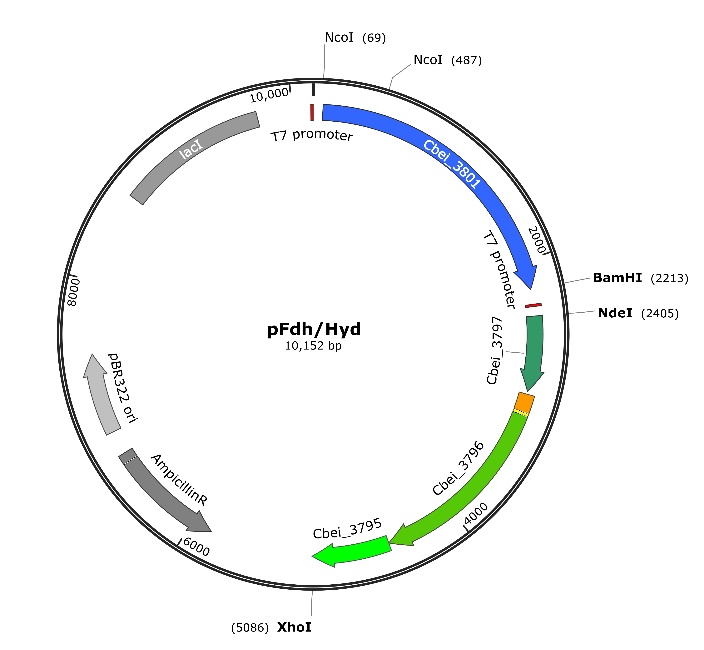


**Figure S5.** Maps of the new plasmids generated in this work. The Cbei_3797, Cbei_3796, Cbei_3795 gene cluster was amplified by PCR from genomic DNA and was only modified to insert an affinity tag (Twin-Strep-Tag, orange) followed by a TEV site (yellow) at the N-terminus of Cbei_3796 (pHyd) or Cbei_3795 (pHyd-3795).

**Supplementary references.**

1 Morra, S., Arizzi, M., Valetti, F. and Gilardi, G. (2016) Oxygen Stability in the New FeFe -Hydrogenase from Clostridium beijerinckii SM10 (CbA5H). Biochemistry. **55**, 5897-5900 https://doi.org/10.1021/acs.biochem.6b00780

2 Chen, Z. J., Lemon, B. J., Huang, S., Swartz, D. J., Peters, J. W. and Bagley, K. A. (2002) Infrared studies of the CO-inhibited form of the Fe-only hydrogenase from Clostridium pasteurianum I: Examination of its light sensitivity at cryogenic temperatures. Biochemistry. **41**, 2036-2043 https://doi.org/10.1021/bi011510o

3 Winkler, M., Senger, M., Duan, J. F., Esselborn, J., Wittkamp, F., Hofmann, E. et al. (2017) Accumulating the hydride state in the catalytic cycle of FeFe -hydrogenases. Nat. Commun. **8**, 7 https://doi.org/10.1038/ncomms16115

4 Sommer, C., Adamska-Venkatesh, A., Pawlak, K., Birrell, J. A., Rudiger, O., Reijerse, E. J. et al. (2017) Proton Coupled Electronic Rearrangement within the H-Cluster as an Essential Step in the Catalytic Cycle of FeFe Hydrogenases. J. Am. Chem. Soc. **139**, 1440-1443 https://doi.org/10.1021/jacs.6b12636

5 Rodriguez-Macia, P., Reijerse, E. J., van Gastel, M., DeBeer, S., Lubitz, W., Rudiger, O. et al. (2018) Sulfide Protects FeFe Hydrogenases From O-2. J. Am. Chem. Soc. **140**, 9346-9350 https://doi.org/10.1021/jacs.8b04339

6 Roseboom, W., De Lacey, A. L., Fernandez, V. M., Hatchikian, E. C. and Albracht, S. P. J. (2006) The active site of the FeFe -hydrogenase from Desulfovibrio desulfuricans. II. Redox properties, light sensitivity and CO-ligand exchange as observed by infrared spectroscopy. J. Biol. Inorg. Chem. **11**, 102-118 https://doi.org/10.1007/s00775-005-0040-2

7 Chongdar, N., Pawlak, K., Rüdiger, O., Reijerse, E. J., Rodriguez-Macia, P., Lubitz, W. et al. (2020) Spectroscopic and biochemical insight into an electron-bifurcating FeFe hydrogenase. J. Biol. Inorg. Chem. **25**, 135-149 https://doi.org/10.1007/s00775-019-01747-1

8 Caserta, G., Adamska-Venkatesh, A., Pecqueur, L., Atta, M., Artero, V., Roy, S. et al. (2016) Chemical assembly of multiple metal cofactors: The heterologously expressed multidomain FeFe -hydrogenase from Megasphaera elsdenii. Biochim. Biophys. Acta-Bioenerg. **1857**, 1734-1740 https://doi.org/10.1016/j.bbabio.2016.07.002

9 Land, H., Sekretareva, A., Huang, P., Redman, H. J., Nemeth, B., Polidori, N. et al. (2020) Characterization of a putative sensory FeFe -hydrogenase provides new insight into the role of the active site architecture. Chem. Sci. **11**, 12789-12801 https://doi.org/10.1039/d0sc03319g

10 Morra, S., Duan, J. F., Winkler, M., Ash, P. A., Happe, T. and Vincent, K. A. (2021) Electrochemical control of FeFe -hydrogenase single crystals reveals complex redox populations at the catalytic site. Dalton Trans. **50**, 12655-12663 https://doi.org/10.1039/d1dt02219a

11 Silakov, A., Kamp, C., Reijerse, E., Happe, T. and Lubitz, W. (2009) Spectroelectrochemical Characterization of the Active Site of the FeFe Hydrogenase HydA1 from Chlamydomonas reinhardtii. Biochemistry. **48**, 7780-7786 https://doi.org/10.1021/bi9009105

12 Corrigan, P. S., Tirsch, J. L. and Silakov, A. (2020) Investigation of the Unusual Ability of the FeFe Hydrogenase from Clostridium beijerinckii to Access an O-2-Protected State. J. Am. Chem. Soc. **142**, 12409-12419 https://doi.org/10.1021/jacs.0c04964

13 Schwarz, F. M., Schuchmann, K. and Muller, V. (2018) Hydrogenation of CO2 at ambient pressure catalyzed by a highly active thermostable biocatalyst. Biotechnol. Biofuels. **11**, 11 https://doi.org/10.1186/s13068-018-1236-3

14 Schuchmann, K. and Muller, V. (2013) Direct and Reversible Hydrogenation of CO2 to Formate by a Bacterial Carbon Dioxide Reductase. Science. **342**, 1382-1385 https://doi.org/10.1126/science.1244758

15 Leo, F., Schwarz, F. M., Schuchmann, K. and Muller, V. (2021) Capture of carbon dioxide and hydrogen by engineered Escherichia coli: hydrogen-dependent CO2 reduction to formate. Appl. Microbiol. Biotechnol. **105**, 5861-5872 https://doi.org/10.1007/s00253-021-11463-z

16 Pinske, C. and Sargent, F. (2016) Exploring the directionality of Escherichia coli formate hydrogenlyase: a membrane-bound enzyme capable of fixing carbon dioxide to organic acid. MicrobiologyOpen. **5**, 721-737 https://doi.org/10.1002/mbo3.365

17 McDowall, J. S., Murphy, B. J., Haumann, M., Palmer, T., Armstrong, F. A. and Sargent, F. (2014) Bacterial formate hydrogenlyase complex. Proceedings of the National Academy of Sciences. **111**, E3948-E3956 https://doi.org/doi:10.1073/pnas.1407927111

18 Wang, S. N., Huang, H. Y., Kahnt, J., Mueller, A. P., Kopke, M. and Thauer, R. K. (2013) NADP-Specific Electron-Bifurcating FeFe -Hydrogenase in a Functional Complex with Formate Dehydrogenase in Clostridium autoethanogenum Grown on CO. J. Bacteriol. **195**, 4373-4386 https://doi.org/10.1128/jb.00678-13

19 Schägger, H. (2006) Tricine-SDS-PAGE. Nature Protocols. **1**, 16-22 https://doi.org/10.1038/nprot.2006.4
